# 16 kDa vasoinhibin binds to integrin alpha5 beta1 on endothelial cells to induce apoptosis

**DOI:** 10.1530/EC-18-0116

**Published:** 2018-04-05

**Authors:** Kazunori Morohoshi, Ryo Mochinaga, Tsukasa Watanabe, Ryojun Nakajima, Toshio Harigaya

**Affiliations:** Department of Life SciencesLaboratory of Functional Anatomy, Faculty of Agriculture, Meiji University, Kawasaki, Japan

**Keywords:** vasoinhibins, integrin alpha5 beta1, integrin beta1, 16 k prolactin, integrin

## Abstract

Many functions of vasoinhibins have been reported, but its receptor has not been clarified yet. Vasoinhibins, 11–18 kDa N-terminal fragments of prolactin, have anti-angiogenic activity and act on endothelial cells to induce apoptosis and to inhibit migration and proliferation, which are opposite to the effects of prolactin. Although vasoinhibins bind to the prolactin receptor, its binding activity is very weak compared to prolactin. Therefore, in this study, we evaluated the binding activity between 16 kDa vasoinhibin and integrin beta1, alpha5 beta1, alpha1 beta1 and alphaV beta3 to identify a specific receptor for vasoinhibins. Moreover, we examined whether 16 kDa vasoinhibin induced apoptosis through integrin beta1 and alpha5 beta1 in endothelial cells. In this study, binding assays and co-immunoprecipitation experiments demonstrated that 16 kDa vasoinhibin could bind strongly to integrin beta1 and alpha5 beta1. Moreover, neutralizing with integrin beta1 and alpha5 beta1 antibody could inhibit 16 kDa vasoinhibin-induced apoptosis in endothelial cells. These findings suggest that vasoinhibins can act on endothelial cells through integrin alpha5 beta1 to induce apoptosis.

## Introduction

Prolactin (PRL) is a 23 kDa peptide hormone that is mainly produced in the anterior pituitary gland. PRL structurally has three disulfide bonds and four alpha helixes (1, 2). N-terminal fragments (11–18 kDa) derived from GH/PRL family hormones have an anti-angiogenic effect, which are produced through the cleavage of some proteases; matrix metalloproteinases (MMPs), bone marrow peptide-1 and cathepsin D (CathD) (3). The fragments were named ‘vasoinhibins’ due to their properties (4). Vasoinhibins (Vi) are generated by proteolytic cleavage within the long-loop structure connecting the third helix and the fourth helix of PRL (5). Vi can act on endothelial cells to induce apoptosis and inhibit proliferation and migration (6).

Although Vi derived from PRL can bind to the PRL receptor, its binding activity is very weak compared to PRL. Hence, we consider that Vi cannot activate the same signal pathway as that of PRL. In addition, it has been reported that Vi exhibits a strong affinity to 52 and 32 kDa peptides (7). Bajou *et al*. (8) reported that Vi could be co-localized with the PAI-1/uPA/uPAR complex and bound to PAI-1 and that the complex was important for inducing anti-angiogenesis. However, a specific receptor for Vi and its precise mechanism are not yet been clarified.

Endostatin, tumstatin and arresten are known as anti-angiogenic factors, which have some properties similar to Vi. Endostatin is cleaved from a type18 collagen by Cath D, B, L and MMPs (9, 10, 11, 12) and tumstatin and arresten are cleaved from a type 4 collagen by MMPs. These enzymes are the same kind of enzymes involved in the generation of Vi from PRL. In addition, it has been reported that a type18 collagen and a type 4 collagen are involved in angiogenesis. Furthermore, it is known that endostatin attenuates MAPK activity to inhibit endothelial cell migration and inhibits eNOS phosphorylation induced by VEGF to suppress the tubular formation of endothelial cells (13, 14) and that tumstatin inhibits an activation of FAK/PI3K/Akt/mTOR to induce apoptosis and arresten activates caspase3 through FAK/p38 MAPK pathway to induce apoptosis.

Endostatin, tumstatin and arresten have a similarity with Vi as described earlier and bind to integrin alpha5 beta1, alpha1 beta1 and alphaV beta3 respectively to induce an anti-angiogenic effect (15). Therefore, we hypothesized that Vi might exhibit anti-angiogenic effects through its receptors integrin alpha5 beta1, alpha1 beta1 and alphaV beta3.

In this study, we tried to evaluate the binding activity between Vi and the integrins to identify a specific receptor for Vi. Moreover, we examined whether Vi induced apoptosis and cell proliferation through integrin alpha5 beta1 in endothelial cells.

## Materials and methods

### Hormones

Recombinant mouse prolactin (mPRL) was obtained from Dr A Parlor (National Hormone and Pituitary Program, Torrance, CA, USA). Human recombinant fibronectin (FN) was purchased from Corning, and Collagen1 (Col1) from human placenta was purchased from Sigma-Aldrich. Recombinant 16 kDa Vi (rVi) was produced in *Escherichia coli* and purified as described previously (16). mPRL, FN, Col1 and rVi were biotinylated using EZ-Link NHS-PEG4 Biotinylation Kit (Thermo Fisher Scientific).

### Cell culture

Human umbilical vein endothelial cells (HUVEC) were purchased from DS Pharma Biomedical (Osaka, Japan). The cells were maintained in Endothelial Growth Medium-2 (EGM-2, Lonza, Basel, Switzerland) prepared according to the manufacturer’s instructions, at 37°C under controlled humidity and 5% CO_2_ atmosphere.

### Binding assay of Vi and integrins

The wells of a 96 well-microtiter plate were coated with integrin beta1, integrin alpha5 beta1, integrin alpha1 beta1 or alphaV beta3 at 100 ng/well and incubated overnight at 4°C. After washing the wells with Delfia PlateWash (PerkinElmer), the wells were blocked by Blocker BSA in TBS (Thermo Fisher Scientific) at room temperature with shaking. Then, biotinylated proteins were added to each well at 0, 10 or 100 nM concentrations and incubated for 3 h at room temperature with shaking. After the incubation, peroxidase-labeled streptavidin was added to each well and reacted for 30 min at room temperature. Subsequently, 1-Step Ultra TMB-ELISA (Thermo Fisher Scientific) was added to each well and incubated for 20 min at room temperature with shaking. Thereafter, the absorbance of each reacted biotinylated protein was measured at 450 nm by a microplate reader (Enspire, Perkin Elmer). Biotinylated FN was used as positive control for integrin beta1, alpha5 beta1 and alphaV beta3 and biotinylated Col1 was used as positive control for integrin alpha1 beta1.

### Co-immunoprecipitation

Co-immunoprecipitation was performed using an immunoprecipitation kit (proteinG, Roche). Biotinylated mPRL or rVi was incubated with a recombinant human integrin alpha5 beta1 on a rotator overnight at 4°C. Mouse anti-human integrin alpha5 beta1 monoclonal antibody (Millipore) was added to proteinG agarose, and the beads–antibody mixture was rotated overnight at 4°C. Mouse IgG was added to proteinG agarose instead of integrin alpha5 beta1 monoclonal antibody as negative control. The antibody coupled beads were blocked by the addition of mouse IgG at 4°C. Then, the integrin alpha5 beta1-biotinylated hormone mixture was added to the antibody coupled beads and rotated for 1 h at 4°C. After the centrifugation at 12,000 ***g*** for 1 min, the supernatant was removed by aspiration, and a wash buffer (150 mM NaCl, 1% Nonidet P40, 0.05% sodium deoxycholate, 50 mM Tris–HCl, pH 7.5) was added to the beads. Thereafter, an elution buffer (125 mM Tris–HCl, 4% SDS, 10% 2-mercaptoethanol, 20% glycerol) was added to the beads, and the beads were boiled for 5 min at 95°C to elute a protein from the beads. The eluted proteins from the beads were utilized for subsequent experiments.

### Western blotting

The proteins eluted from the beads were electrophoresed on 15% SDS-polyacrylamide gel and then the separated proteins were transferred onto the PVDF membrane. The membrane was blocked with EzBlock Chemi (ATTO, Tokyo, Japan). Subsequently, the membrane was incubated with an anti-prolactin rabbit antibody overnight at 4°C. After the incubation, the membrane was incubated with a peroxidase-labeled anti-rabbit IgG antibody (Vector Laboratories, Burlingame, CA,USA) for 30 min at room temperature. After washing the membrane three times with TBS-Tween, the immunoreactive bands were detected by Immobilon western chemiluminescent HRP substrate (Millipore).

### TUNEL assay

A TUNEL assay was performed using In Situ Cell Death Detection Kit, Fluorescein (Roche) to detect apoptosis in cell culture according to the kit manufacturer’s protocol. HUVEC were seeded onto CORNING BIOCOAT cellware rat tail collagen type1, 8-Well Culture Slides (Corning) at a density of 1.0 × 10^4^ cells/well and cultured overnight. The cells were treated with 6.0 × 10^4^ µg/well integrin beta1 or integrin alpha5 beta1 neutralizing antibody for 2 h and then stimulated with 3.2 µg/well rVi for 24 h. After the stimulation, the cells were fixed with 4% paraformaldehyde for 1 h. Then, the cells were permeabilized with 0.1 % Triton-X in PBS for 5 min on ice and blocked by a blocking reagent containing 3% bovine serum albumin (Sigma-Aldrich), and 20% normal goat serum (Vector Laboratories), for 30 min at room temperature. The TUNEL reaction solution supplied from In Situ Cell Death Detection Kit was added to the cells and incubated for 1 h at 37°C. After washing the cells three times with PBS, FLUOROSHIELD Mounting Medium with 4,6-diamidino-2-phenylindole (DAPI, Immuno BioScience Corp., Mukilteo, WA, USA) was applied and a cover slip was mounted. The stained cells were observed using FLUOVIEW FV1000 (OLYMPUS). Five fields were selected randomly from each well under a 100× field and TUNEL-positive cells (%) in the field were quantified using ImageJ software (17).

### BrdU assay

BrdU assay was performed using BrdU Immunohistochemistry Kit (Merck Millipore, Germany) to confirm whether Vi affects cell proliferation in cell culture. HUVEC were seeded onto CORNING BIOCOAT cellware rat tail collagen type1, 8-Well Culture Slides (Corning) at a density of 1.0 × 10^4^ cells/well and cultured overnight. The cells were treated with 6.0 × 10^4^ µg/well integrin beta1 or integrin alpha5 beta1 neutralizing antibody for 2 h and then stimulated with 3.2 µg/well rVi for 24 h. After the stimulation, the cells were fixed with 70% ethanol for 30 min at room temperature. Then, the cells were permeabilized with denaturing solution for 30 min at room temperature and blocked by a blocking solution for 30 min at room temperature. Detector Antibody, biotinylated BrdU antibody, was added to the wells and incubated for 1 h at room temperature. Then, DyLight 594 Streptavidin (Vector Laboratories) was added to the wells and incubated for 2 h under a light-shielded condition. After washing the cells three times with PBS, FLUOROSHIELD Mounting Medium with 4,6-diamidino-2-phenylindole (DAPI, Immuno BioScience Corp.) was applied and a cover slip was mounted. The stained cells were observed using FLUOVIEW FV1000 (OLYMPUS). Five fields were selected randomly from each well under a 100× field and BrdU-positive cells (%) in the field were quantified using ImageJ software (18).

### Statistical analysis

Experimental data were expressed as mean ± s.d. *T* tests were used for statistical analysis (*P* < 0.05 was considered statistically significant).

## Results

### Vasoinhibin binds with integrins

**Figure 1:**
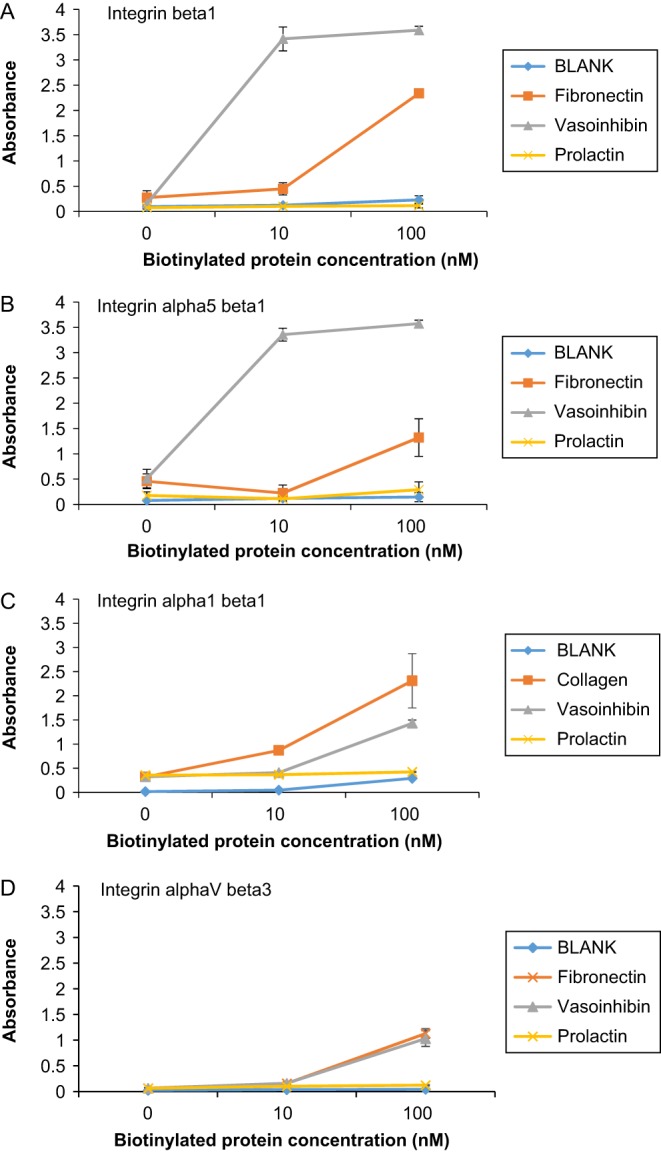
Vasoinhibin binds integrins 0, 10, 100 nM biotinylated proteins were added to 96-well plate coated with integrin beta1 (A), integrin alpha5 beta1 (B), alpha1 beta1 (C) or alphaV beta3 (D) at 100 ng/well concentration. Absorbance was measured 3 h after addition of biotinylated proteins. (A) Absorbance of biotinylated Vi incubated with integrin beta1 was significantly higher than that of biotinylated FN (*P* < 0.01). (B) Absorbance of biotinylated Vi incubated with integrin alpha5 beta1 was significantly higher than that of biotinylated FN (*P* < 0.01). (C) Absorbance of biotinylated Vi incubated with integrin alpha1 beta1 was significantly higher than that of BLANK (*P* < 0.01). (D) Absorbance of biotinylated Vi incubated with integrin alphaV beta3 was significantly higher than that of BLANK (*P* < 0.01). Data represent mean ± s.d. of 3 independent experiments, double asterisks indicate significant differences (*P* < 0.01).

We assessed the binding capacity of Vi to integrin beta1, alpha5 beta1, alpha1 beta1 and alphaV beta3 by using the binding assay. The absorbance of biotinylated rVi with integrin beta1 was higher than that of biotinylated FN. However, no significant difference in biotinylated PRL was observed (Fig. 1A).

The absorbance of biotinylated rVi with integrin alpha5 beta1 was higher than that of biotinylated FN, but no significant difference in biotinylated PRL was observed (Fig. 1B). The absorbance of biotinylated rVi with integrin alpha1 beta1 and alphaV beta3 was higher than that of BLANK (Fig. 1C and D). These results suggest that the binding capacity of integrin alpha5 beta1 was highest among examined integrins.

**Figure 2:**
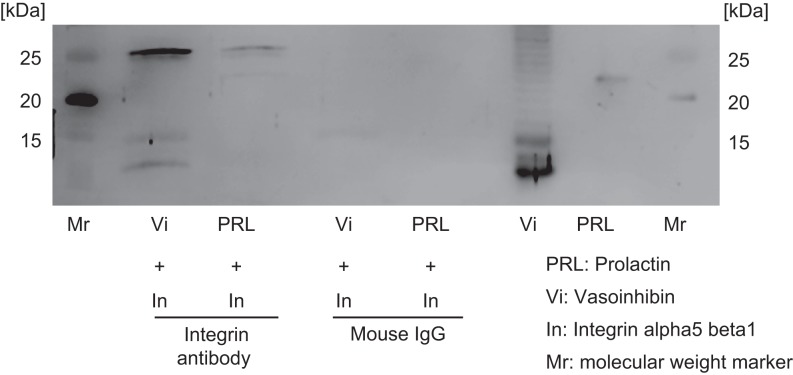
Vasoinhibin binds integrin alpha5 beta1 rVi and mPRL after immunoprecipitation of integrin alpha5 beta1 were detected by western blot using an anti-prolactin antibody. Mouse IgG was used for immunoprecipitation as a negative control. rVi was detected after immunoprecipitation, but mPRL was not detected.

Since rVi binds to integrin alpha5 beta1 more strongly than the other integrins, integrin alpha5 beta1 was applied for co-immunoprecipitation experiment and neutralizing experiment. A co-immunoprecipitation experiment was performed using rVi and integrin alpha5 beta1 to confirm the binding reaction of Vi to integrin alpha5 beta1. rVi was detected in the mixture of rVi and integrin alpha5 beta1 by western blot using integrin alpha5 beta1 antibody (Fig. 2).

### Integrin beta1 and integrin alpha5 beta1 neutralizing antibody inhibit the apoptotic effect of vasoinhibin but not cell proliferation

**Figure 3:**
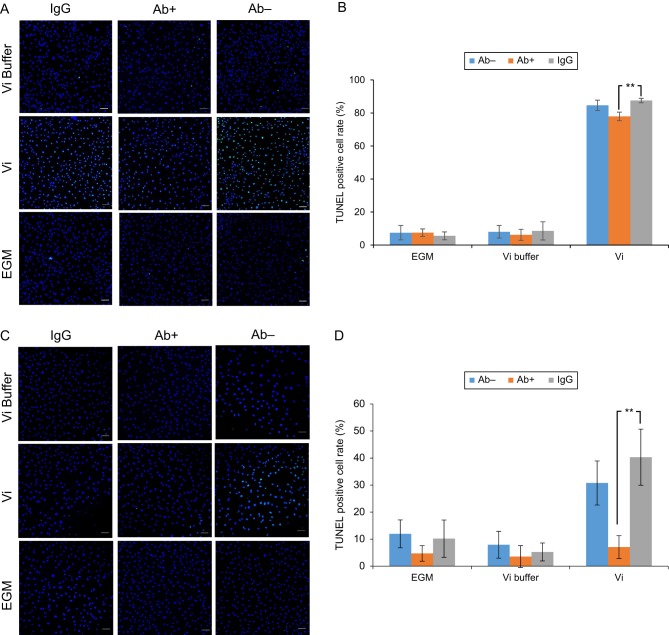
Integrin antibody inhibits an apoptotic effect of vasoinhibin incubating HUVEC with Vi for 24 h increases the TUNEL-positive cell rate. Integrin beta1 antibody (A, B) or alpha5 beta1 antibody (C, D) was added to HUVEC before incubation with Vi. (A and C) Immunofluorescence images showed that the integrin antibodies decreased TUNEL-stained cell (green). Nuclei are stained blue. Scale bar indicates 100 µm. (B) Integrin beta1 antibody decreased the TUNEL-positive cell rate induced by Vi (*P* < 0.01). (D) Integrin alpha5 beta1 antibody also decreased TUNEL-positive cell rate induced by Vi (*P* < 0.01). Data represent mean ± s.d. of 3 independent experiments, double asterisks indicate significant differences (*P* < 0.01).

To explore whether integrin beta1 was involved in the apoptosis activity of Vi in endothelial cells, we performed the TUNEL assay after HUVEC was treated with rVi and the integrin beta1 antibody. In the absence of the antibody, the ratio of TUNEL-positive cells in the rVi group was significantly higher than that in the control group. Conversely, in the presence of the antibody, no significant difference was observed between the rVi group and the control group (Fig. 3A and B).

In addition, we treated the integrin alpha5 beta1 antibody to examine whether integrin alpha5 beta1 is involved in the apoptosis of Vi. In the absence of the antibody, the ratio of the TUNEL-positive cells in the rVi group was significantly higher than that in the control group. Conversely, in the presence of the antibody, no significant difference was observed between the rVi group and the control group (Fig. 3C and D).

**Figure 4:**
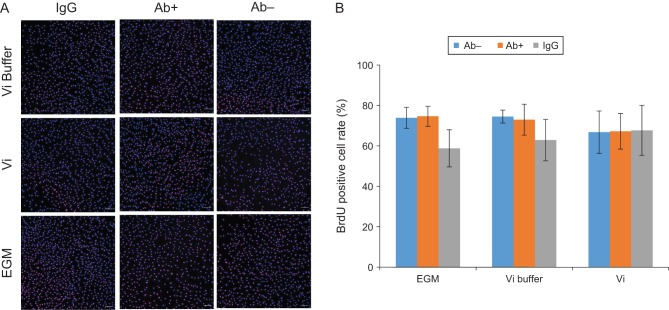
Incubating HUVEC with Vi for 24 h did not affect the BrdU-positive cell rate. Integrin alpha5 beta1 antibody was added to HUVEC before incubation with Vi. (A) BrdU-positive cells were identified by immunohistochemistry (red). Nuclei are stained blue. Scale bar indicates 100 µm. (B) Vi did not affect the BrdU-positive cell rate in this experimental condition. Data represent mean ± s.d. of 3 independent experiments.

After HUVEC was treated with rVi and the integrin alpha5 beta1 antibody, we performed the BrdU assay to investigate whether integrin alpha5 beta1 is involved in the proliferation activity of Vi in endothelial cells. In this study, we showed Vi did not affect the cell proliferation rate in HUVEC under the 24-h incubation condition (Fig. 4).

## Discussion

Vi have some similar properties to endostatin, tumstatin and arresten, which can bind to some integrins. Integrins are comprising noncovalent and heterodimeric complexes, and each complex consists of an alpha and a beta-subunit. In mammals, integrins have 18 alpha and 8 beta subunits, which can form 24 unique integrin complexes (19). The integrin beta1 subunit is the most common, and it can form the complexes with integrin alpha subunits. Thus, we analyzed the binding activity between Vi and integrin beta1 and showed that Vi and integrin beta1 formed a complex. Then, we examined which heterodimeric integrins Vi can bind to, because it is known that integrins exist naturally as dimers. Endostatin acts on endothelial cells to induce an anti-angiogenic effect through integrin alpha5 beta1 (14) and tumstatin and arresten induce an anti-angiogenic effect through integrin alphaV beta3 and alpha1 beta1 respectively. In this study, therefore, we investigated the binding activity between the integrins and Vi and revealed that Vi can induce an anti-angiogenic effect on endothelial cells through integrin alpha5 beta1.

The binding assay suggests that Vi binds to integrin alpha5 beta1, alpha1 beta1 and alphaV beta3. Co-immunoprecipitation also suggests that Vi binds to integrin alpha5 beta1. It has been reported that Vi has the opposite effect of PRL (3). Therefore, we speculated that a specific receptor for Vi could exist. In this study, we showed that Vi can form a complex with integrin alpha5 beta1, but PRL cannot. Thus, this suggests that the unique effects of Vi, including anti-angiogenesis, arise through integrin alpha5 beta1.

The immune neutralization by integrin alpha5 beta1 and beta1 antibody revealed that these antibodies attenuate apoptosis induced by Vi. This result shows that Vi induced apoptosis through integrin alpha5 beta1 in endothelial cells. Endostatin acts on endothelial cells through integrin alpha5 beta1 to regulate Bcl-2 and caspase-3 expression, and then induce apoptosis (20). Vi also acts on HUVEC to regulate caspase 3, 8 and 9, and then induces apoptosis (21). Accordingly, integrin alpha5 beta1 is involved in a signal transduction of Vi.

In addition, Vi has anti-proliferation effects on endothelial cells (6, 22). We also investigated whether integrin alpha5 beta1 antibody attenuated anti-cell proliferation activity induced by Vi. However, we did not confirm anti-cell proliferation activity on HUVEC in all experimental conditions. Our experimental conditions might not be suitable for confirming anti-proliferation activity of Vi.

Clapp *et al*. (7) reported that proteins 32 kDa and 52 kDa have a high affinity to Vi. However, the molecular weight of integrin is not equal to these proteins, so we hypothesize that another protein is involved in the formation of Vi-integrin complex and that Vi forms a complex with multiple proteins. Bajou *et al*. (8) reported that Vi could be co-localized with the PAI-1/uPA/uPAR complex and bound to PAI-1 and that the complex was important for inducing anti-angiogenesis. In addition, Tarui *et al*. (23) reported that uPAR bound directly to integrin to regulate signal transduction. Consequently, we speculate that Vi, PAI-1, uPA, uPAR and integrin form a complex and that this complex plays a role in anti-angiogenesis. Further investigation is required to reveal the precise connection between Vi, integrin alpha5 beta1 and PAI-1/uPA/uPAR complex.

Vi has been identified in various tissues including pituitary tissue and endothelial cells (4, 24). In this study, we showed that Vi binds to integrin alpha5 beta1, and although we did not verify which tissue Vi is produced in, it is likely that Vi is produced in tissues other than pituitary tissue. The reason for this assumption is that integrin alpha5 beta1 expresses on a basolateral surface and connects to cells or ECM. So, if Vi is produced in pituitary tissue, it is unlikely that would bind to integrin alpha5 beta1 because the Vi would be secreted into blood circulation. Also there is a possibility that Vi derived from ectopic PRL binds to integrin expressed on the same or nearby cell through an autocrine or paracrine mechanism. We also speculate that the effects of Vi arise locally in various tissues that express Vi, regardless of the Vi concentration in the blood.

## Declaration of interest

The authors declare that there is no conflict of interest that could be perceived as prejudicing the impartiality of the research reported.

## Funding

This work is supported by JSPS KAKENHI, Grant Number 23591092.
